# Mineralization of the herbicide swep by a two-strain consortium and characterization of a new amidase for hydrolyzing swep

**DOI:** 10.1186/s12934-020-1276-9

**Published:** 2020-01-07

**Authors:** Long Zhang, Ping Hang, Xiyi Zhou, Chen Dai, Ziyi He, Jiandong Jiang

**Affiliations:** 1 Department of Microbiology, Key Lab of Microbiology for Agricultural Environment, Ministry of Agriculture, College of Life Sciences, Nanjing Agricultural University, Nanjing, 210095 China; 2 College of Life Sciences, Huaibei Normal University, Huaibei, 235000 China; 3 Jiangsu Provincial Key Lab for Organic Solid Waste Utilization, Nanjing Agricultural University, Nanjing, 210095 China

**Keywords:** *Comamonas* sp. SWP-3, *Alicycliphilus* sp. PH-34, Consortium, Swep, 3,4-Dichloroaniline, Degradation

## Abstract

**Background:**

Swep is an excellent carbamate herbicide that kills weeds by interfering with metabolic processes and inhibiting cell division at the growth point. Due to the large amount of use, swep residues in soil and water not only cause environmental pollution but also accumulate through the food chain, ultimately pose a threat to human health. This herbicide is degraded in soil mainly by microbial activity, but no studies on the biotransformation of swep have been reported.

**Results:**

In this study, a consortium consisting of two bacterial strains, *Comamonas* sp. SWP-3 and *Alicycliphilus* sp. PH-34, was enriched from a contaminated soil sample and shown to be capable of mineralizing swep. Swep was first transformed by *Comamonas* sp. SWP-3 to the intermediate 3,4-dichloroaniline (3,4-DCA), after which 3,4-DCA was mineralized by *Alicycliphilus* sp. PH-34. An amidase gene, designated as *ppa*, responsible for the transformation of swep into 3,4-DCA was cloned from strain SWP-3. The expressed Ppa protein efficiently hydrolyzed swep and a number of other structural analogues, such as propanil, chlorpropham and propham. Ppa shared less than 50% identity with previously reported arylamidases and displayed maximal activity at 30 °C and pH 8.6. Gly449 and Val266 were confirmed by sequential error prone PCR to be the key catalytic sites for Ppa in the conversion of swep.

**Conclusions:**

These results provide additional microbial resources for the potential remediation of swep-contaminated sites and add new insights into the catalytic mechanism of amidase in the hydrolysis of swep.

## Introduction

Carbamate pesticides are a new class of broad-spectrum pesticides that function as insecticides, acaricides and herbicides [1]. These compounds are highly selective, efficient and widely used in agriculture, forestry and animal husbandry. Over 1000 types of carbamate pesticides have been developed, and their use has exceeded that of organophosphorus pesticides, with the sales of carbamate herbicides being only second to pyrethroid pesticides [1]. However, the extensive use of carbamate herbicides leads to the presence of carbamate herbicide residues in the environment, threating ecosystems and human health [2–4]. Since the 1970s, the use of carbamate herbicides has increased every year.

Among these compounds, swep (methyl *N*-(3,4-dichlorophenyl) carbamate) is an excellent herbicide that kills weeds by interfering with metabolic processes and inhibiting cell division at the growth point [5, 6]. Due to the large amount of swep used (1–1.5 kg of 25% wettable powder per 667 m^2^), swep residues in soil and water not only cause environmental pollution but also accumulate through the food chain, ultimately pose a threat to human health [7]. Swep was reported to be highly toxic to fish [8], and in rats the mortality rate reached 50% when the orally administered concentration of swep reached 522 mg/kg. Furthermore, the mortality of carp after 48 h was more than 50% when the concentration of swep exceeded 2.6 mg/L [8]. Swep can be absorbed through the human respiratory tract, digestive tract and skin, subsequently inhibiting the activity of acetylcholinesterase, causing clinical manifestations that are similar to those of organophosphorus pesticides [8]. 3,4-Dichloroaniline (3,4-DCA) was reported to be the primary metabolite of swep and also showed toxic effects on mammals and fish, as well as on the human immune system [9, 10]. The environmental fates of swep and its metabolite 3,4-DCA has attracted much attention.

Microbial biodegradation is the primary pathway for the removal of organic pollutants from environments [11–14]. Numerous bacterial strains, such as species with in the genus of *Variovorax* [15, 16], *Sphingomonas* sp. strain Y57 [17] and *Achromobacter* sp. strain ANB-1 [18], have been isolated and characterized from various habitats that are capable of mineralizing 3,4-DCA. Eleven genes that responsible for the aniline derivative oxygenation (*dcaQTA1A2BR*) and catechol ortho-cleavage catabolism (*ccdRCFDE*) have been also widely reported [16, 18]. However, to date, no studies on the biotransformation of swep have been reported and enzymes involving in the degradation of swep have also not been characterized previously.

In this study, the mineralization of swep by a consortium consisting of *Comamonas* sp. SWP-3 and *Alicycliphilus* sp. PH-34 was investigated. A new amidase (Ppa) capable of transforming swep to 3,4-DCA was characterized, and the key amino acid residues in Ppa required for catalysis were investigated. The results of this study will add new insights into the biodegradation of carbamate herbicides.

## Materials and methods

### Chemical compounds and medium

J&K Chemical Co., Ltd. (Shanghai, China) was the source for obtaining of all chemical compounds (purity ≥ 98%). TaKaRa Biotechnology Co., Ltd. (Dalian, China) provided all of the molecular biology reagents. Using mediums were derived from Zhang et al. [19] and Hang et al. [20].

### Bacterial strains, plasmids and culture conditions

Using primers, bacterial strains and plasmids are shown in Additional file 1: Table S1 and Table 1, respectively. The concentrations of used antibiotics were carried out as previously described [21].

**Table 1:** Strains and plasmids used in this study

Strains and plasmids	Characteristics	Source or reference(s)
Strains		
*E. coli* DH5*α*	F^**−**^*recA1 endA1 thi*-*1 supE44 relA1 deoR Δ(lacZYA*-*argF)U169Φ* 80d*lacZΔ* M15	Invitrogen
*E. coli* BL21(DE3)	F^**−**^*ompT hsd*S_B_(r_B_^**−**^m_B_^**−**^) *dcm gal λ*(DE3)	Invitrogen
*Comamonas* sp. SWP-3	Wild type, Str^r^, capable of degrading various herbicides	This study
*Comamonas* sp. SWP-3 M	The *ppa* gene-knockout mutant of strain SWP-3 with *ppa* gene, Str^r^	This study
*Comamonas* sp. SWP-3C	Mutant of strain SWP-3 with *ppa* gene restored, Str^r^	This study
*Alicycliphilus* sp. PH-34	Wild type, Amp^r^, capable of degrading various aniline derivatives	This study
Plasmids		
pMD19-T	TA cloning vector; Amp^r^	TaKaRa
pEX18-Gm	Suicide vector; Gm^r^	TaKaRa
pBBR1MCS-2	Broad host-range vector; kanamycin^r^ (Km^r^)	Lab store
pRK2013	Conjugation helper plasmid	Lab store
pET-29a (+)	Expression vector; Km^r^	Invitrogen
pMCS2-ppa	pBBR1MCS-2 containing the *ppa* gene; Km^r^	This study
pET-ppa	pET-29a (+) containing the *ppa* gene; Km^r^	This study
pEX18-ppa	pEX18-Gm containing the *ppa* gene; Gm^r^	This study
pMD19-ppa	pMD19-T containing the *ppa* gene; Amp^r^	This study
pMD19-ppa-T1	pMD19-T containing the *ppa*-*T1* gene; Amp^r^	This study
pMD19-ppa-T2	pMD19-T containing the *ppa*-*T2* gene; Amp^r^	This study

### Strain isolation and characterization

The swep-contaminated soil was obtained from a pesticide factory in Nantong, China. 100 g of the soil was added into 1 L of mineral salt medium (MSM) containing 30 mg/L swep and incubated at 30 °C for 7 days. The enrichment culture (15 mL) was subsequently inoculated into 100 mL of fresh MSM (containing 30 mg/L of swep) and cultured as previously described [21]. Enrichment solution with stable swep degradation activity was diluted and spread onto Luria–Bertani (LB) agar plates containing 30 mg/L of swep and incubated at 30 °C for 5 days. Different forms of individual colonies on the plates were picked and inoculated into 1/4 R2A medium (supplemented with 30 mg/L of swep or 3,4-DCA) and then checked for their degradation abilities after 5 days. Instead, a strain (designated SWP-3) showed significantly swep-transforming ability and another strain (designated PH-34) possessed the capability of catabolizing 3,4-DCA were isolated using swep or 3,4-DCA as the substrates. The isolates were identified as previously described [20, 21].

### Synergistic catabolism of swep by the two-strain consortium

Strains SWP-3 and PH-34 were individually cultured in LB broth, and the cells were collected and washed twice with sterilized MSM medium. The OD_600_ of each strain resuspended in MSM medium was adjusted to 1.0, after which equal volumes of each strain were mixed. For synergistic degradation, the mixture was inoculated into 100 mL of MSM medium (containing 30 mg/L of swep or 3,4-DCA) at a final OD_600_ of 0.2 and then was incubated at 30 °C with shaking at 160 rpm on a rotary shaker. For individual degradation assays, strain SWP-3 or PH-34 was inoculated into 100 mL of MSM medium (containing 30 mg/L of swep or 3,4-DCA) at final OD_600_ values of 0.2 and incubated under the same conditions. Samples were withdrawn periodically, and the concentrations of swep/3,4-DCA were determined by HPLC. Spectrophotometer (UV-2450, SHIMADZU, Japan) was used to determine the cell growth at OD_600_. All of these experiments were replicated three times.

### Substrate spectra of strain SWP-3 and strain PH-34

The degradation capacity of strain SWP-3 for other structural analogues of swep (such as linuron, diuron, carbofuran, carbaryl, diflubenzuron, propanil, propham and chlorpropham) were studied. Strain SWP-3 (0.4 mL, OD_600_ = 1.0) was inoculated into 20 mL of MSM medium containing 30 mg/L of various of substrates. The cultures were incubated at 30 °C, 160 rpm for 5 days, after which the concentrations of the substrates were detected by HPLC. To investigate the degradation substrates by strain PH-34, aniline derivatives, including *m*-chloroaniline, *m*-methylaniline, *p*-methylaniline, 3-bromoaniline, 3-chloro-4-methylaniline, 3-chloro-4-methoxybenzenamine, 4-bromoaniline, 4-bromo-3-chloroaniline, *p*-chloroaniline and 4-isopropylaniline were tested as described above.

### Chemical analysis

The analysis of swep, its metabolite 3,4-DCA and analogues of swep and 3,4-DCA, were performed by HPLC, the details for which are described as before [18, 21].

### Cloning of the swep hydrolase gene

Genomic DNA was extracted and purified from strain SWP-3 using a commercial genomic DNA extraction kit (Solarbio Science and Technology Company) according to the manufacturers’ instructions and was subsequently partially digested with *Sau3*AI. The shotgun method was used to clone the hydrolase gene and performed as described previously [21]. The transformants were plated onto LB agar containing 100 mg/L ampicillin and 0.5 mM 4-acetaminophenol (a structural analogue of swep) and then incubated at 37 °C for 12 h. Brown colonies potentially harboring the target amidase were selected for exhibiting the capacity to transform 4-acetaminophenol into the brown product 4-aminophenol [22]. Positive clones were further tested for their ability to hydrolyze swep by HPLC, and then sequenced immediately. Analyzing the sequence results was performed as previously described methods [21].

### Gene expression and purification of Ppa

A suspected swep hydrolase gene (designated *ppa*) was cloned and Ppa was expressed as an *N*-terminal fusion to a hexa-histidine tag (His-6) to facilitate purification. The primers used to amplify the *ppa* gene are listed in Additional file 1: Table S1. PCR product was connected with pET-29a (+), and then transformed into *E. coli* BL21. The purification procedure of Ppa is described in Additional file 1 in detail.

### Enzymatic characterization of Ppa

A standard enzymatic assay was performed in 3 mL of Tris–HCl buffer (100 mM, pH 8.0) containing 10 mg/L swep. The reaction was initiated by adding the purified Ppa to a final concentration of 0.16 µg/mL and was incubated at 30 °C for 30 min before being stopped by adding 3 mL of dichloromethane. The determination of thermal stability, optimal reaction temperature, optimum pH and the effects of different metal ions are described in Additional file 1 in detail.

To determine the substrate spectrum of Ppa and estimate its kinetic values to different substrates, a detailed procedure for which is also included in Additional file 1.

### Genetic disruption and complementation

To disrupt the *ppa* gene by gene targeting, two DNA fragments (approximately 730 bp flanking each end of *ppa*) were generated by PCR using the primer pairs PP1-F/PP1-R and PP2-F/PP2-R (Additional file 1: Table S1). Subsequently, the two fragments were joined using the primer pair PP1-F/PP2-R (Additional file 1: Table S1) by overlap extension PCR. The resulting product was then cloned into the XbaI and BamHI sites of the plasmid pEX18-Gm (Takara) to yield pEX18-ppa, which was then conjugated into strain SWP-3 from *E. coli* DH5*α* with the help of the plasmid pRK2013. The transconjugants were selected on LB plates supplemented with streptomycin (Str) and gentamicin (Gm). The mutant SWP-3 M with the disrupted *ppa* gene was checked by PCR. For the complementation of the disrupted *ppa* gene, the *ppa* gene was amplified with the primer pair PPA1-F/PPA1-R (Additional file 1: Table S1) and ligated into the corresponding sites of the broad-host-range plasmid pBBR1-MCS2 [23], yielding pBBR1-ppa. The plasmid pBBR1-ppa was then mobilized into the *ppa*-disrupted mutant through electroporation to generate the complemented strain SWP-3C. The swep degradation abilities of strains SWP-3, SWP-3M and SWP-3C were assessed as described above.

### RT-qPCR

Strain SWP-3 was pre-cultured in MSM medium with different substrates (0.3 mM of swep, propanil, chlorpropham, propham, glucose or 4-acetaminophenol) at 30 °C for 12 h, after which total RNA was extracted as the way described before [18]. The RT-qPCR procedure is described in Additional file 1 in detail.

### Identification of the key catalytic residues of Ppa for swep hydrolysis

To generate the Ppa variants with no hydrolytic activity against swep, sequential error prone PCR was performed. Unlike high-fidelity PCR, dCTP (2 or 8 mM), dTTP (2 or 8 mM), Mg^2+^ (5, 6, or 7.5 mM) and Mn^2+^ (0.05, 0.2, or 0.6 mM) were added to the PCR system (50 µL) to improve the possibility of error. An XbaI site was incorporated into the primers (Additional file 1: Table S1) to facilitate directional cloning of the amplified PCR product into pMD-19T. The plasmid pMD19T-ppa was used as the template and was used at a 10-fold dilution compared to the normal amount used for PCR amplification of *ppa*. The resulting product (*ppa*-*T*) was then cloned into the XbaI site of the plasmid pMD-19T to yield pMD-19T-ppa-T. Subsequently, pMD19T-ppa and pMD-19T-ppa-T were transformed into competent *E. coli* DH5*α* cells, which were plated onto LB plates containing 100 mg/L ampicillin (Amp) and 0.5 mM 4-acetaminophenol to construct a variant library of Ppa. Clones showing no ability to form brown halos compared to the wild-type strain were screened. Clones showing the ability to hydrolyze swep were selected for further testing via enzymatic assays.

## Results

### Isolation and identification of strains involved in swep degradation

Isolation of a pure culture that can mineralize swep from the enrichment culture failed. Instead, bacterial strains that capable of hydrolyzing swep to 3,4-DCA (namely strain SWP-3, deposited in CCTCC under the number CCTCC AB 2019366) or mineralizing 3,4-DCA (namely strain PH-34, deposited in CCTCC under the number CCTCC AB 2019367) were isolated. Both the SWP-3 and PH-34 strains are non-spore-forming, motile with terminal flagella and rod-shaped (0.8–0.9 × 1.7–1.8 µm and 0.7–0.8 × 1.3–1.4 µm, respectively) (Additional file 1: Fig. S1). Phylogenetic analysis based on the 16S rRNA gene sequences showed that strain SWP-3 clustered within the *Comamonas* species and formed a subclade with *Comamonas terrigena* NBRC 13299^T^ (99.8% identity) (Additional file 1: Fig. S2), while strain PH-34 clustered within the *Alicycliphilus* genus and formed a subclade with *Alicycliphilus denitrificans* K601^T^ (100% identity) (Additional file 1: Fig. S3). Thus, strains SWP-3 and PH-34 were preliminarily identified as *Comamonas* sp. and *Alicycliphilus* sp., respectively.

The substrate spectra of strains SWP-3 and PH-34 were investigated. In addition to swep, strain SWP-3 could also metabolize propanil, chlorpropham and propham. However, PH-34 could degrade aniline compounds such as *m*-chloroaniline, *m*-methylaniline, 4-bromo-3-chloroaniline and *p*-chloroaniline.

### Synergistic biodegradation of swep by the two-strain consortium

**Fig. 1:**
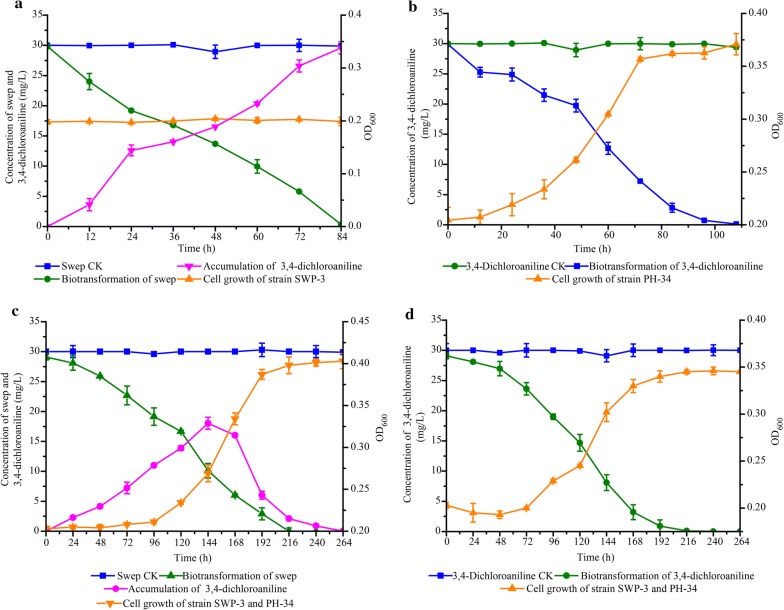
**a** Degradation of swep by *Comamonas* sp. SWP-3; **b** degradation of 3,4-dichloroaniline by *Alicycliphilus* sp. PH-34; **c** degradation of swep by the consortium comprising of *Comamonas* sp. SWP-3 and *Alicycliphilus* sp. PH-34; **d** degradation of 3,4-dichloroaniline by the consortium comprising of *Comamonas* sp. SWP-3 and *Alicycliphilus* sp. PH-34

When swep was degraded by strain SWP-3 alone, 30 mg/L of swep was efficiently removed in 84 h, with one intermediate product observed that accumulated without further transformation, even after 110 h of incubation (Fig. 1a). No growth of strain SWP-3 was observed, showing that it could not use swep as a sole carbon source for growth. When strain PH-34 was incubated with 30 mg/L of 3,4-DCA as the sole carbon source, the compound was completely degraded within 5 days, and the cell density of strain PH-34 increased significantly (Fig. 1b). When swep was degraded by the consortium consisting of both the SWP-3 and PH-34 strains, 30 mg/L of swep was completely degraded, and the intermediate 3,4-DCA initially appeared (within 144 h) before disappearing at 264 h. Cell growth of the consortium was observed (Fig. 1c). For the degradation of 3,4-DCA by the consortium, 30 mg/L of 3,4-DCA was completely degraded in 216 h (Fig. 1d) (the inoculum of strain PH-34 in the synergistic biodegradation test was half of that used in the individual biodegradation test). Cell growth was also observed in this assay, primarily due to the growth of strain PH-34 (Fig. 1c, d).

### Cloning of the amidase gene *ppa*

A positive clone which can degrade 4-acetaminophenol (producing a brown color) was obtained from about 18,000 transformants, and the inserted fragment (4919 bp) was sequenced. Based on the ORF analysis and BLASTp results, one ORF encoding a protein with moderate identity to amidases was identified. The transformant harboring the gene also showed the ability to transform swep. Therefore, this ORF, namely *ppa* was the swep hydrolase gene. Sequence analysis showed that the *ppa* gene is 1488 bp in length and encodes a protein with 495 amino acid residues. Ppa showed the highest identity to acylamidase (*Rhodococcus* sp. TA37; K9NBS6.1) (33.5%) and the putative amidase AF_1954 (*Archaeoglobus fulgidus* sp. DSM 4304; O28325.1) (32.9%) (available from the NCBI Swissprot protein database).

Based on the selection for a double-crossover event, a *ppa*-disrupted SWP-3 M mutant was obtained. Compared with the wild type strain SWP-3 (Additional file 1: Fig. S4A), the cell suspension assay showed that the mutant strain SWP-3M strain lost the capacity to transform swep (Additional file 1: Fig. S4C), whereas the *ppa*-complemented strain SWP-3C (harboring the plasmid pBBR1-ppa) was restored for the ability to transform swep (Additional file 1: Fig. S4B). These results showed that *ppa* is the only gene responsible for swep hydrolysis in strain SWP-3.

The gene *ppa* was constitutively transcribed, which was not notably enhanced by its substrates (swep, propanil, chlorpropham, propham and 4-acetaminophenol) compared to glucose (Additional file 1: Fig. S5), this was in accordance with the corresponding accumulation of their products (data not shown).

### The catalytic characteristics of Ppa

**Fig. 2:**
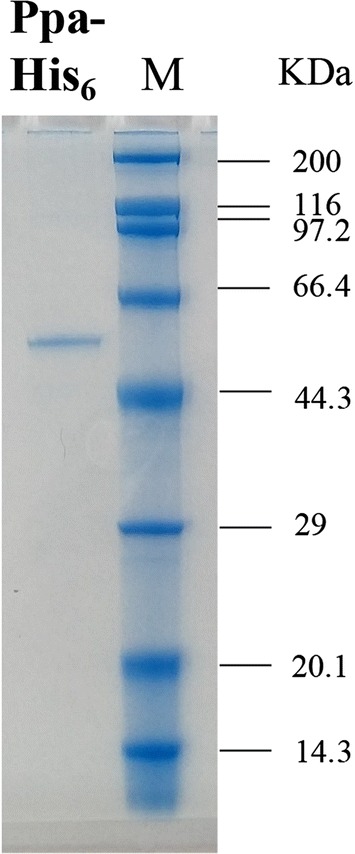
SDS-PAGE of the recombinant Ppa purified from *E. coli BL*21 (DE3) (pET-ppa) by Ni^2+^-NTA. *Ppa-His6* the purified recombinant Ppa; *M* low molecular protein marker

**Fig. 3:**
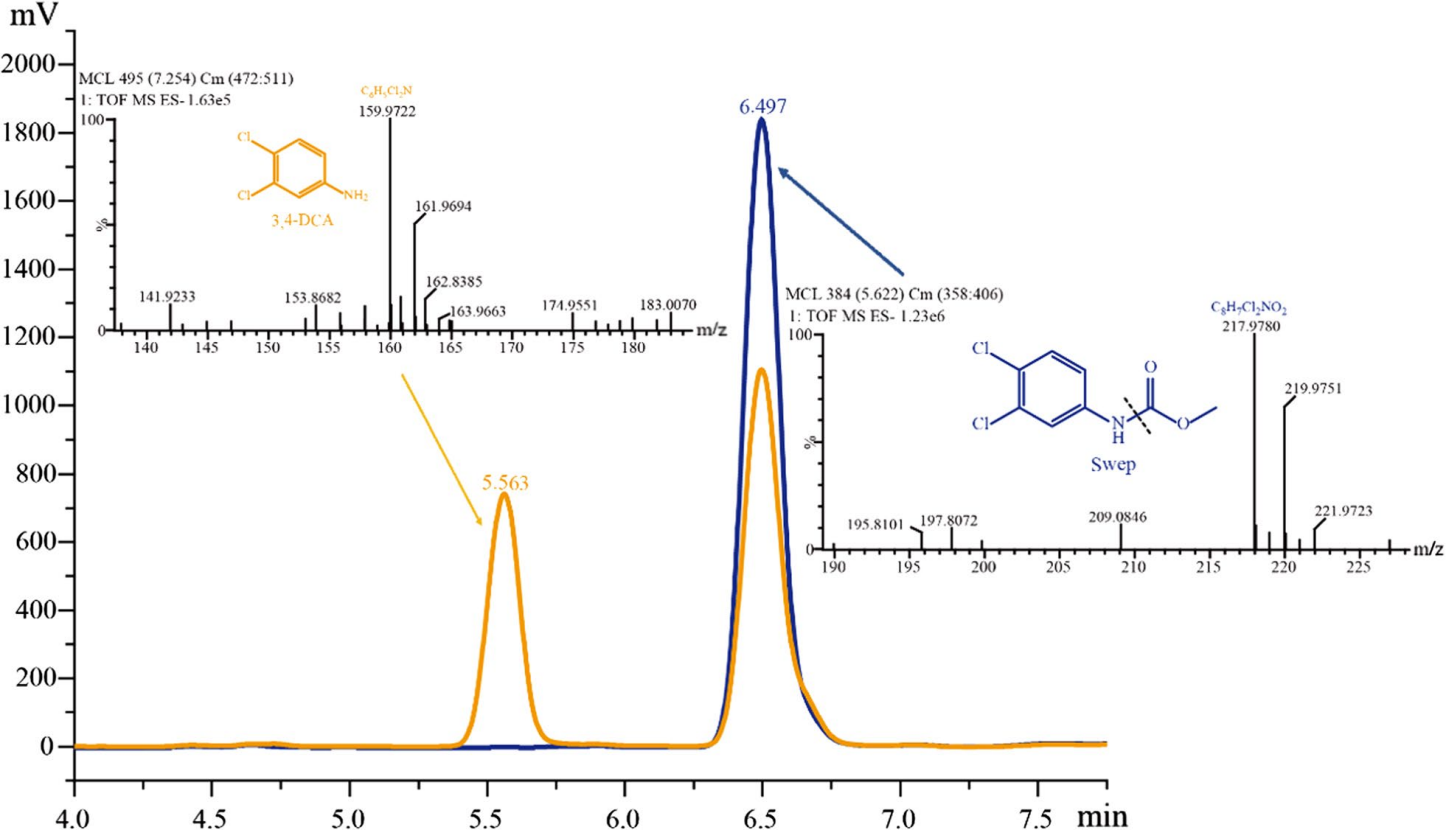
LC–MS analyses of the metabolite produced during swep degradation by recombinant Ppa purified from *E. coli* BL21 (DE3) (pET-ppa). The HPLC profiles of swep [Rt (min) = 6.497] and the metabolite 3,4-DCA [Rt (min) = 5.563] are indicated. The mass spectra of swep {m/z = 217.9780 [M − H]^−^} (right) and 3,4-DCA {m/z = 159.9722 [M − H]^−^} (left) are also shown

Recombinant Ppa was successfully expressed and purified. The purified enzyme appeared as a single band with an approximately molecular mass of 55 kDa in the SDS-PAGE analysis (Fig. 2). Ppa was further confirmed to catalyze the hydrolysis of swep to 3,4-DCA (Fig. 3) as well as the hydrolysis of some other herbicides, such as propanil, chlorpropham and propham (Additional file 1: Figs. S6, S7, S8). The kinetics values of Ppa for swep, propanil, chlorpropham and propham are summarized in Table 2. Propanil appeared to be the best substrate for Ppa, with *K*_m_ and *k*_cat_ values of 1.51 μM and 219.90 s^−1^, respectively.

**Table 2:** Kinetic constants of Ppa for swep, propanil, chlorpropham and propham

Formula	*K*_m_ (μM)	*k*_cat_ (s^−1^)	Catalytic efficiency *k*_cat_/*K*_m_ (μM^−1^ s^−1^)
	10.40 ± 1.20	14.58 ± 2.50	1.40
	1.51 ± 0.30	219.90 ± 8.56	145.63
	55.22 ± 5.23	0.99 ± 0.11	0.02
	140.90 ± 11.32	0.73 ± 0.04	0.01

Values are the means ± standard deviations from three experiments

The effects of different factors on Ppa activity have been well investigated. Ppa showed maximal activity at 30 °C, and retained over 70% of its relative activity at 25–55 °C (Additional file 1: Fig. S9A). Ppa retained the highest activity at pH 8.6 and showed relatively high activity at pH values ranging from 7.0 to 10.0 (Additional file 1: Fig. S9B). In addition, Al^3+^, Mn^2+^, Co^2+^, Cu^2+^, Fe^3+^, Cd^2+^ and Zn^2+^ were observed to significantly inhibit Ppa activity, and Ni^2+^, Mg^2+^ and Ca^2+^ inhibited approximately 20–30% of Ppa activity at a concentration of 1 mM (Additional file 1: Fig. S9C).

### Identification the key catalytic residues for Ppa in the conversion of swep

**Fig. 4:**
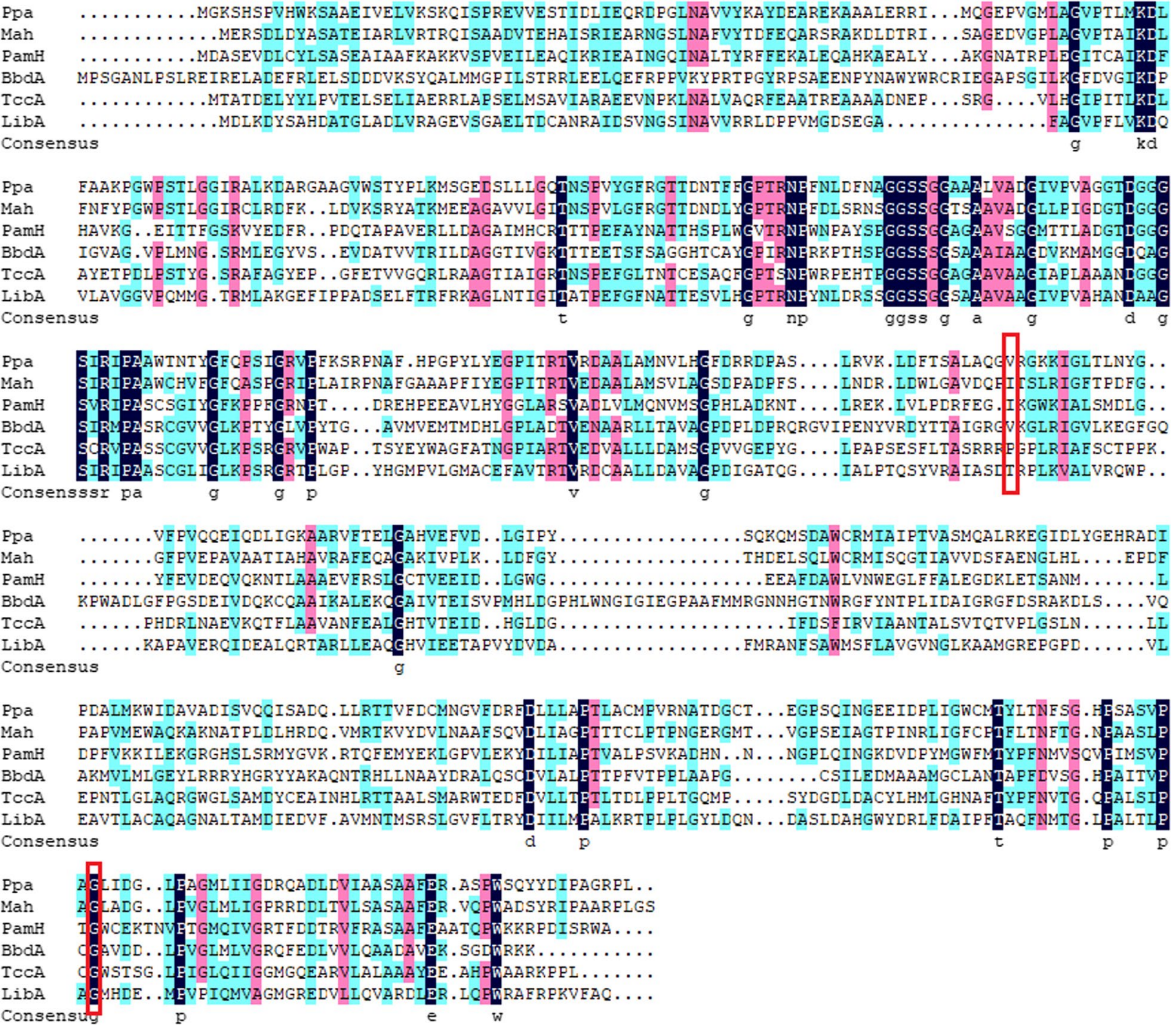
Alignment of amino acid sequences of Ppa and other 5 biochemically characterized amidases from GenBank. Identical amino acid residues are marked in black; similar residues are marked in pink. The amino acid residue sits of Ppa that were mutant by error-PCR are marked by red boxs

By performing sequential error prone PCR, two clones that could not form brown halos around colonies were obtained from approximately 15,000 transformants. HPLC analysis revealed that these two clones lost the ability to degrade swep. The corresponding mutated genes *ppa*-*T1* and *ppa*-*T2* were then sequenced in triplicate. Compared with the wild-type Ppa protein, the Ppa-T1 and Ppa-T2 mutant enzymes had individual amino acid residue mutations of Gly449Asp and Val266Glu, respectively. These results show that Gly449 and Val266 are key catalytic residues for Ppa in the conversion of swep. Alignment of the amino acid sequences of Ppa and other 5 biochemically characterized amidases (Mah, PamH, BbdA, TccA and LibA) (Fig. 4) revealed high conservation of the Gly/Ser-rich motif (GGSS[GS]G) and that Gly449 is a conserved residue in these enzymes.

## Discussion

In this study, a swep-mineralizing consortium was obtained from an enrichment culture. Although strain SWP-3 could not grow when swep was used as the sole carbon source, it could survive in the enrichment culture, suggesting that strain SWP-3 might have used some small aliphatic metabolites produced from other strains in the enrichment culture that could mineralize 3,4-DCA. This hypothesis was confirmed by the isolation of a 3,4-DCA-mineralizing strain (*Alicycliphilus* sp. PH-34), which could use 3,4-DCA as a sole carbon and energy source for growth. More interestingly, though species with in the various genus (such as *Variovorax*, *Sphingomonas* and *Achromobacter*) are capable of mineralizing 3,4-DCA, no species have been reported possessing the 3,4-DCA degradation ability within the genus of *Alicycliphilus*. Degradation of xenobiotics by the microbial are complicated and rarely completely degraded by a single strain [18]. Most of them need to be completely degraded by the synergistic action of microbial consortium. The consortium tends to have more degradative functions and higher degradation efficiency than single strains [24–26]. Thus, in this study, the consortium comprising strains SWP-3 and PH-34 could efficiently and completely mineralize swep, providing a good candidate for the bioremediation of swep-contaminated sites.

**Fig. 5:**
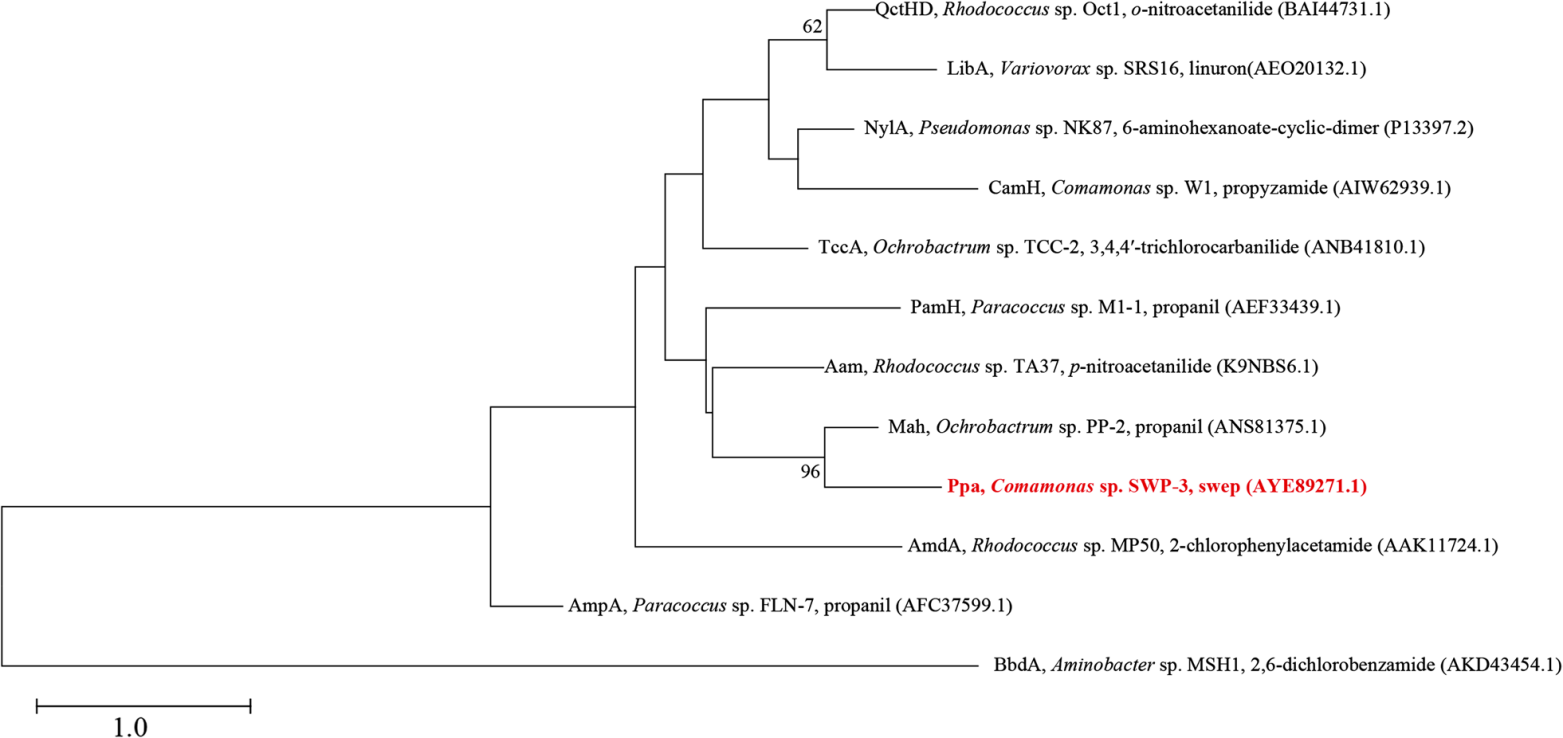
A neighbor-joining phylogenic tree constructed based on the amino acid sequences of Ppa (bold and red) and biochemically characterized amidases. The substrates and their Genbank accession numbers are in line with each amidase, respectively. The phylogenetic neighbor joining tree was constructed using MEGA (version 7.0) and was bootstrapped 1000 times (> 50% are shown at branching points). The bar represents 1.0 amino acid difference per site

The identified Ppa enzyme shares low amino acid identities (28.9 to 33.5%, Swissprot protein database) with other biochemically characterized amidases. Ppa also showed relatively low identity (27 to 50%, on amino acid level) with amidases reported to be able to degrade xenobiotics. For example, Ppa exhibited the highest identity (49.8%) with Mah (the propanil hydrolase, ANS81375.1) [18]; a 34.6% identity with PamH (propanil hydrolase, AEF33439.1) [27]; a 30.5% identity with TccA (triclocarban hydrolase, ANB41810.1) [28]; a 30% identity with BbdA (2,6-dichlorobenzamide hydrolase, AKD43454.1) [29]; a 28.3% identity with LibA (linuron hydrolase, AEO20132.1) [16]; and a 28% identity with CamH (propyzamide hydrolase, AIW62939.1) [30]. The neighbor-joining phylogenic tree based on the amino acid sequence of Ppa and the functionally characterized amidases (capable of degrading xenobiotics) and combine with the high conservation of the Gly/Ser-rich motif (GGSS[GS]G) and the catalytic triad (Ser–Ser–Lys) (Fig. 4) indicated that Ppa is an arylamidase in the amidase signature (as) enzymes family (Fig. 5).

Amidases play key roles in the initial degradation of a wide range of amide xenobiotics [21, 22, 31–34]. To date, diverse amidases involved in the biotransformation of xenobiotics have been reported in many different genera. AmpA/PamH, DmhA and Mah (all for the hydrolysis of propanil) were identified from the genera *Paracoccus* [22, 27], *Sphingomonas* [35] and *Ochrobactrum* [21], respectively. LibA and TccA2 were identified from *Variovorax* [16] and *Diaphorobacter* [18] and are responsible for the initial degradation of linuron. BbdA was identified from *Aminobacter* and initiates the degradation of 2,6-dichlorobenzamide [36]. TccA was identified from *Ochrobactrum* and is involved in the hydrolysis of triclocarban [28]. To the best of our knowledge, no amidases have been reported that initiate the degradation of swep. In this study, Ppa was identified as the functional enzyme involved in swep-transformation and was also shown to hydrolyze some other carbamate herbicides (propham and chlorpropham) and the amide herbicide (propanil). The discovery of the amidase Ppa provides a good candidate for the study of the catalytic mechanism of amidases.

## Conclusion

In this study, a consortium consisting of two bacterial strains, *Comamonas* sp. SWP-3 and *Alicycliphilus* sp. PH-34, was enriched from a contaminated soil sample and shown to be capable of mineralizing swep. A novel swep transforming amidase gene, *ppa*, has been cloned from *Comamonas* sp. SWP-3, and the enzyme, Ppa, has been well characterized. This is the first detail report on the biodegradation of swep. These results showed that a bacterial consortium can contain catabolically synergistic species for swep mineralization and our findings provide additional microbial resources for the potential remediation of swep-contaminated sites and add new insights into the catalytic mechanism of amidase in the hydrolysis of swep.

### Nucleotide sequence accession number

The 16S rRNA and *ppa* gene sequences of strain SWP-3 were deposited at DDBJ/ENA/GenBank under the accession numbers of MH819768 and MH822147, respectively. The 16S rRNA gene sequence of strain PH-34 was deposited at DDBJ/ENA/GenBank under accession number of MH819769.

## Supplementary information

**Additional file 1: Figure S1.** The transmission electron micrographs of strains SWP-3 and PH-34. **Figures S2, S3.** The phylogenetic relationship of strains SWP-3 and PH-34, respectively. **Figure S4.** Degradation curve of swep by the wild type strain, complement strain and the mutant strain. **Figure S5.** RT-qPCR analysis of the transcription of *ppa*. **Figures S6, S7, S8.** HPLC–MS analysis of propanil, chlorpropham and propham hydrolyzed by Ppa, respectively. **Figure S9.** Effects of temperature, pH value and metal ions on the activities of the purified recombinant Ppa. **Table S1.** Primers that were used in this study.

## Data Availability

All materials described within this manuscript, and engineered strains are available on request.
