# Supplementary material for: Mineralization of the herbicide swep by a two-strain consortium and characterization of a new amidase for hydrolyzing swep
Source: Microb Cell Fact. 2020 Jan 7;19:4. doi: 10.1186/s12934-020-1276-9 (PMC6945715; doi:10.1186/s12934-020-1276-9)
Supplement: Supplementary file 1 — Additional file 1: Figure S1. The transmission electron micrographs of strains SWP-3 and PH-34. Figures S2, S3. The phylogenetic relationship of strains SWP-3 and PH-34, respectively. Figure S4. Degradation curve of swep by the wild type strain, complement strain and the mutant strain. Figure S5. RT-qPCR analysis of the transcription of ppa. Figures S6, S7, S8. HPLC–MS analysis of propanil, chlorpropham and propham hydrolyzed by Ppa, respectively. Figure S9. Effects of temperature, pH value and metal ions on the activities of the purified recombinant Ppa. Table S1. Primers that were used in this study. [file 12934_2020_1276_MOESM1_ESM.docx]

**Mineralization of the herbicide swep by a two-strain consortium and characterization of a new amidase for hydrolyzing swep**

Long Zhang^1, 2^, Ping Hang^1^, Xiyi Zhou^1^, Chen Dai^1^, Ziyi He^1^, Jiandong Jiang^1, 3, *^

^1^Department of Microbiology, Key Lab of Microbiology for Agricultural Environment, Ministry of Agriculture, College of Life Sciences, Nanjing Agricultural University, 210095, Nanjing, China

^2^College of Life Sciences, Huaibei Normal University, Huaibei 235000, China

^3^Jiangsu Provincial Key Lab for Organic Solid Waste Utilization, Nanjing Agricultural University, Nanjing, 210095, China

**Running title:** Degradation of swep by a bacterial consortium

**Key words:** *Comamonas* sp. SWP-3; *Alicycliphilus* sp. PH-34; consortium; swep; 3,4-dichloroaniline; degradation

All correspondence should be addressed to Dr. Jiandong Jiang.

E-mail: jiang_jjd@njau.edu.cn; Tel.: +86-25-84399726; Fax: +86-25-84396314

**Figure S1.** Transmission electron micrographs of the negatively stained cells of strain SWP-3 and strain PH-34 that had been grown on LB agar at 30 °C for 12 hours. A, strain SWP-3, Bar, 1.0 µm; B, strain PH-34, Bar, 0.5 µm.


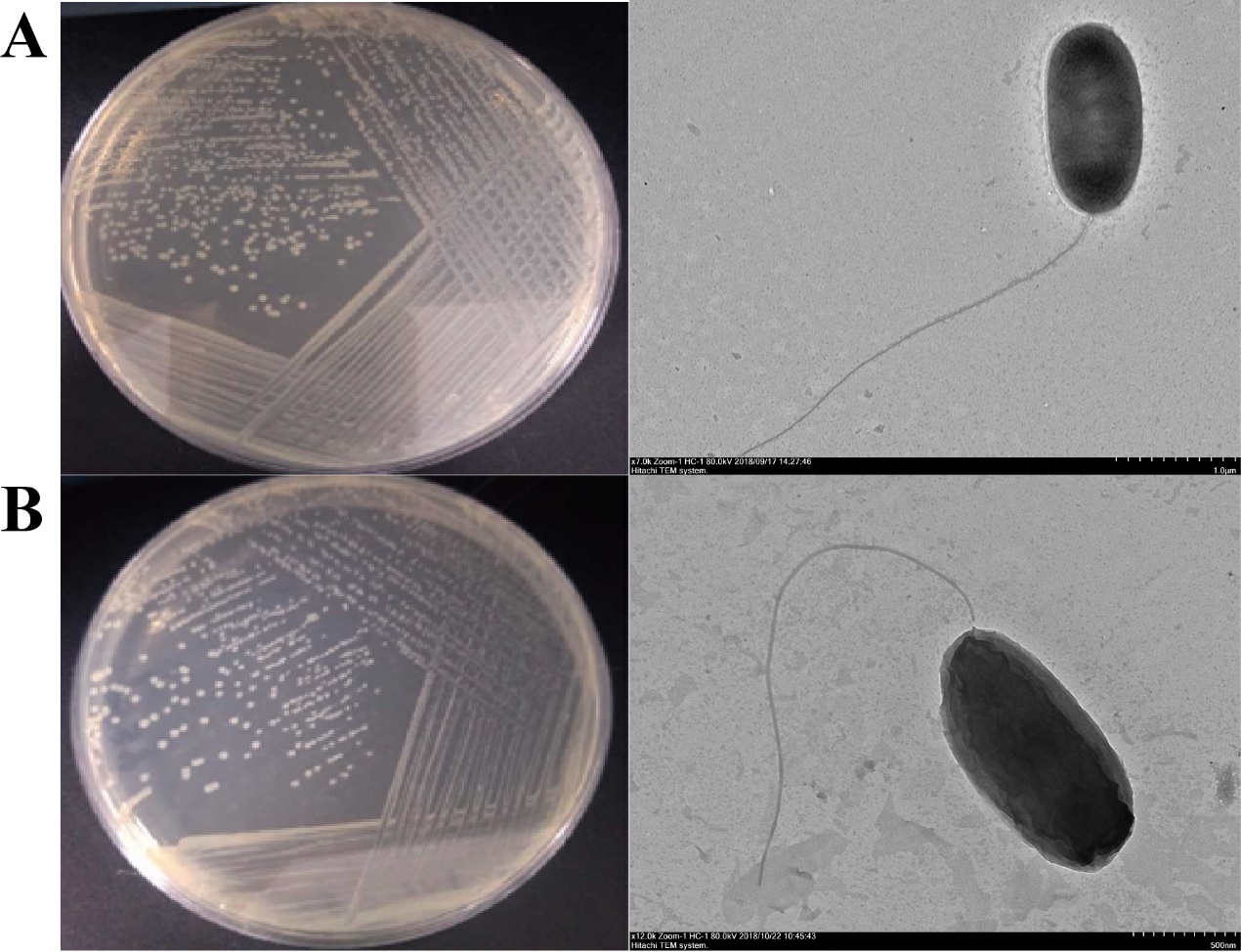


**Figure S2.** Neighbor-joining tree based on 16S rRNA gene sequences shows the phylogenetic relationship of strain SWP-3 among the genus *Comamonas*. Bootstrap values expressed as percentages of 1,000 replications are given at branching points. Only bootstrap values > 50% are depicted. The type strain *Novosphingobium subterraneum* DSM 12447^T^ served as the outgroup. Bar, 2% sequence divergence.


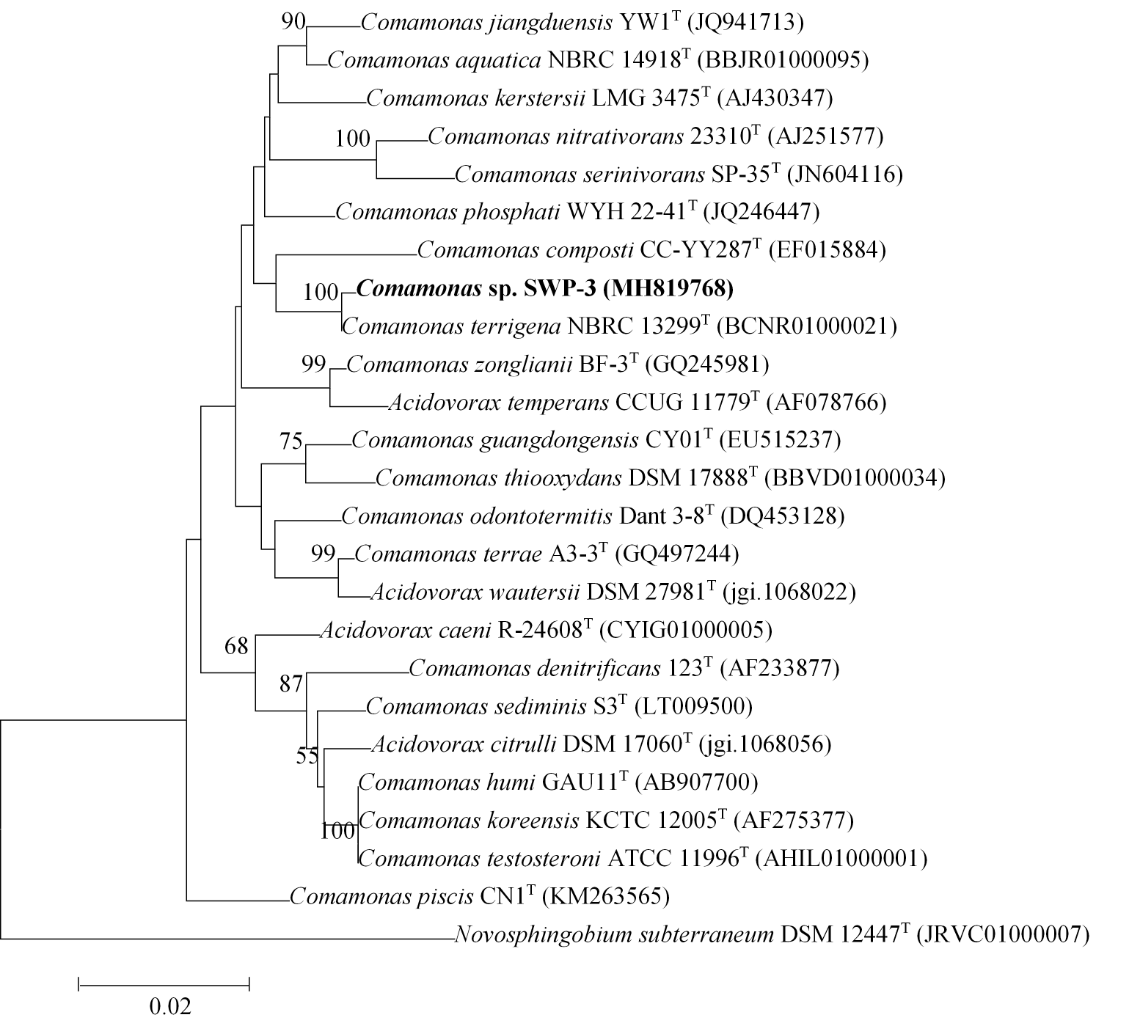


**Figure S3.** Neighbor-joining tree based on 16S rRNA gene sequences shows the phylogenetic relationship of strain PH-34 among the genus *Alicycliphilus*. Bootstrap values expressed as percentages of 1,000 replications are given at branching points. Only bootstrap values > 50% are depicted. The type strain *Novosphingobium subterraneum* DSM 12447^T^ served as the outgroup. Bar, 2% sequence divergence.


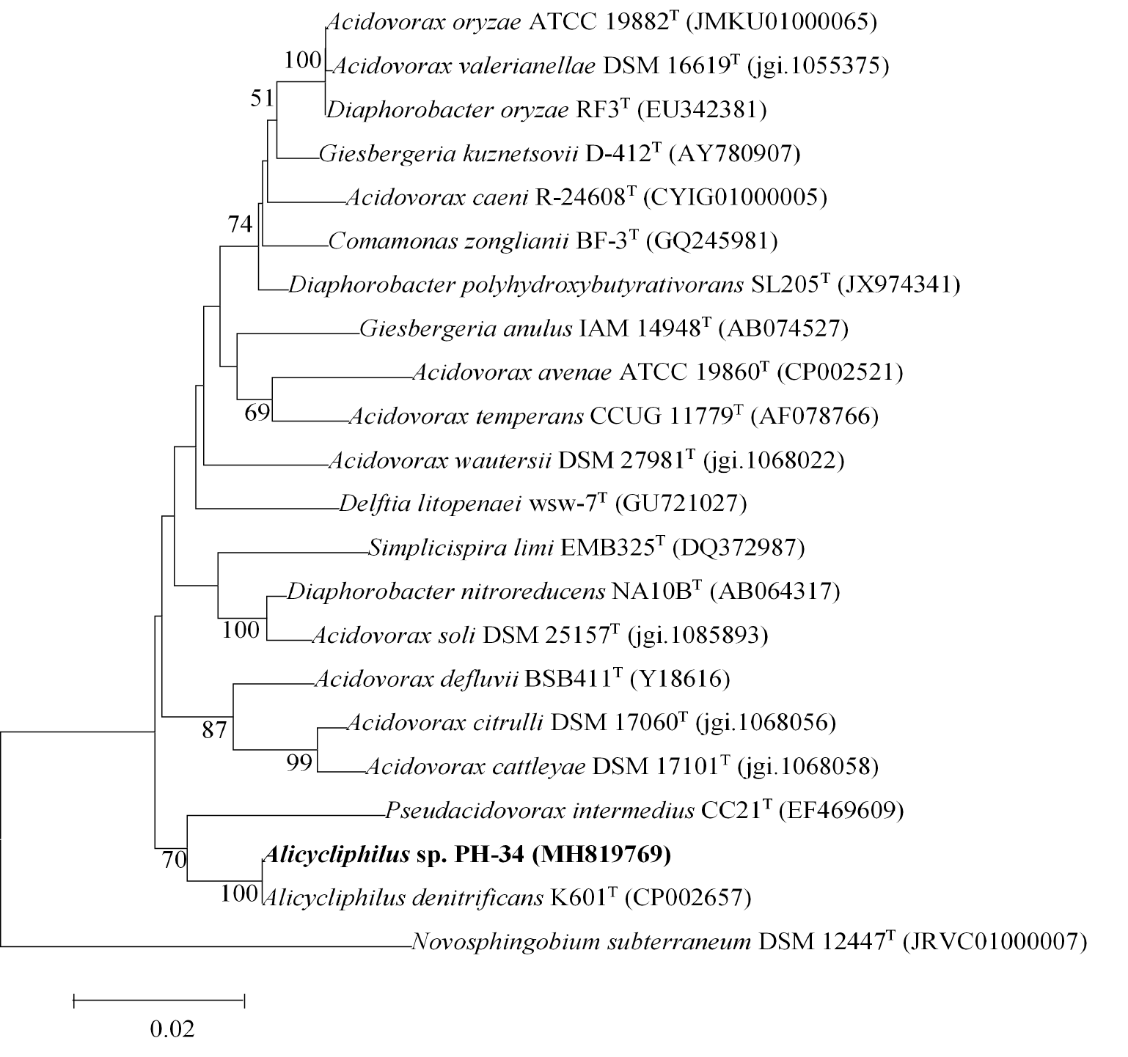


**Figure S4.** Degradation of swep by the wild type *Comamonas* sp. SWP-3 (A), the complement strain *Comamonas* sp. SWP-3C (B) and the mutant *Comamonas* sp. SWP-3M (C).


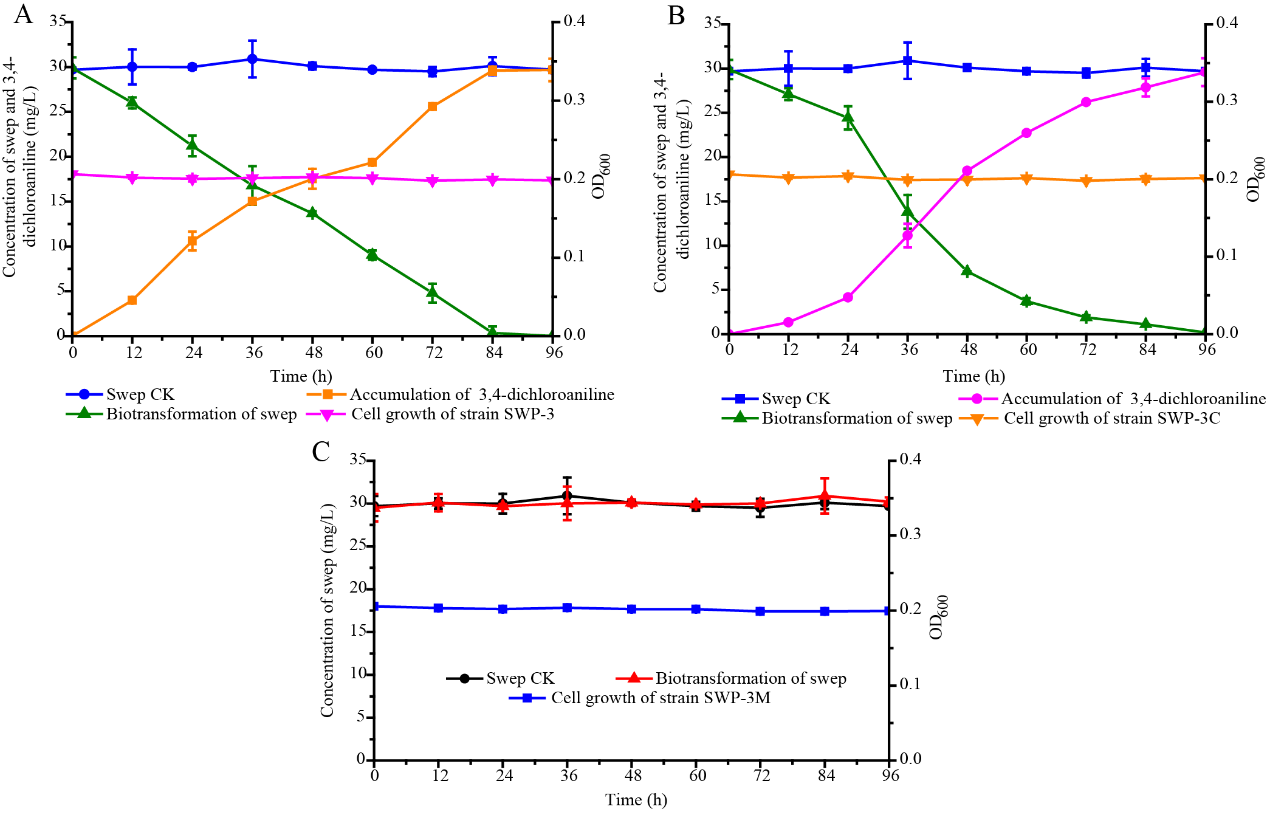


**Figure S5.** RT-qPCR analysis of the transcription of *ppa* in strain SWP-3 upon induction by different substrates. The 16S rRNA gene was used as an internal control.


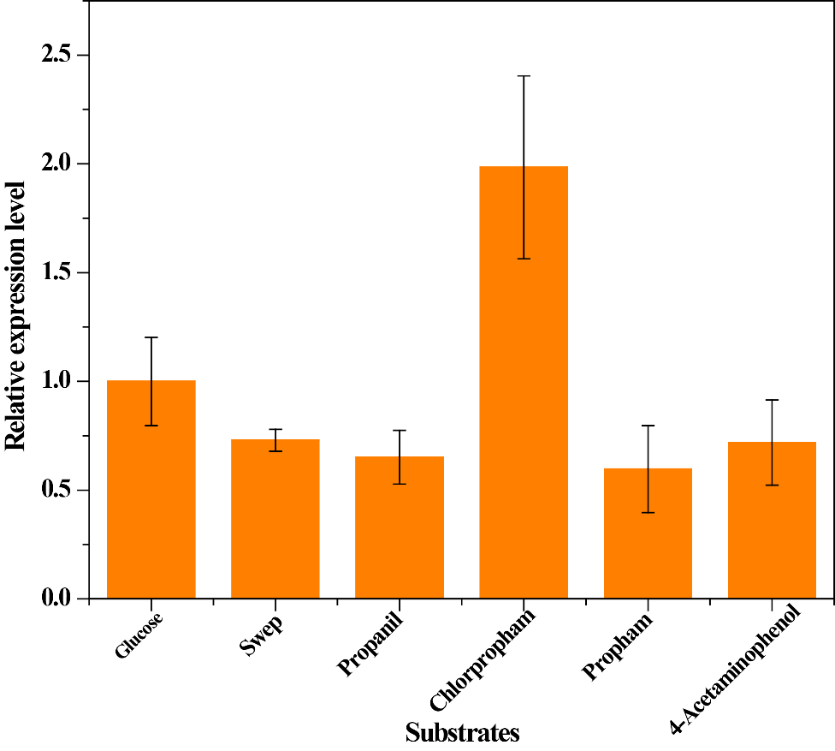


**Figure S6.** LC-MS analyses of the metabolite produced during propanil degradation by the purified recombinant Ppa. The HPLC profiles of propanil [Rt (min)=5.850] and the metabolite 3,4-DCA [Rt (min)= 5.543] are indicated. The mass spectra of propanil {m/z=215.9990 [M−H]^−^} (right) and 3,4-DCA {m/z=159.9734 [M−H]^−^} (left) are also shown.


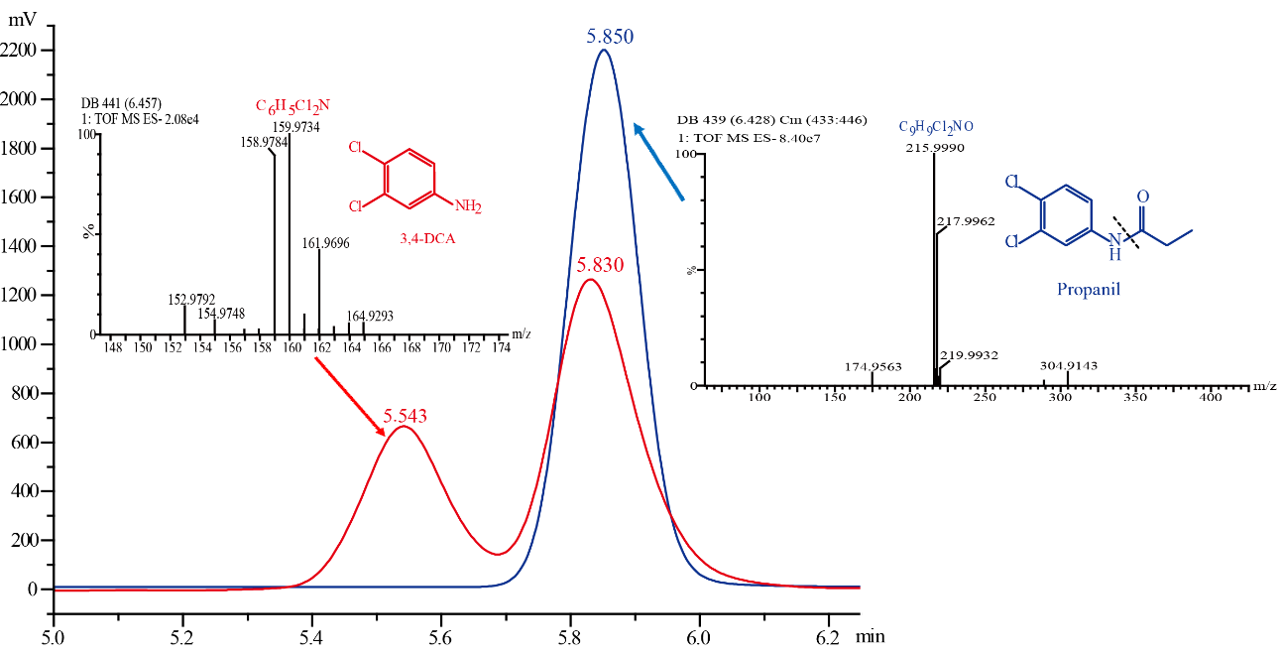


**Figure S7.** LC-MS analyses of the metabolite produced during chlorpropham degradation by the purified recombinant Ppa. The HPLC profiles of chlorpropham [Rt (min)=11.820] and the metabolite *m*-chloroaniline [Rt (min)= 5.590] are indicated. The mass spectra of chlorpropham {m/z=212.0470 [M−H]^−^} (up) and *m*-chloroaniline {m/z=126.0101 [M−H]^−^} (down) are also shown.

**
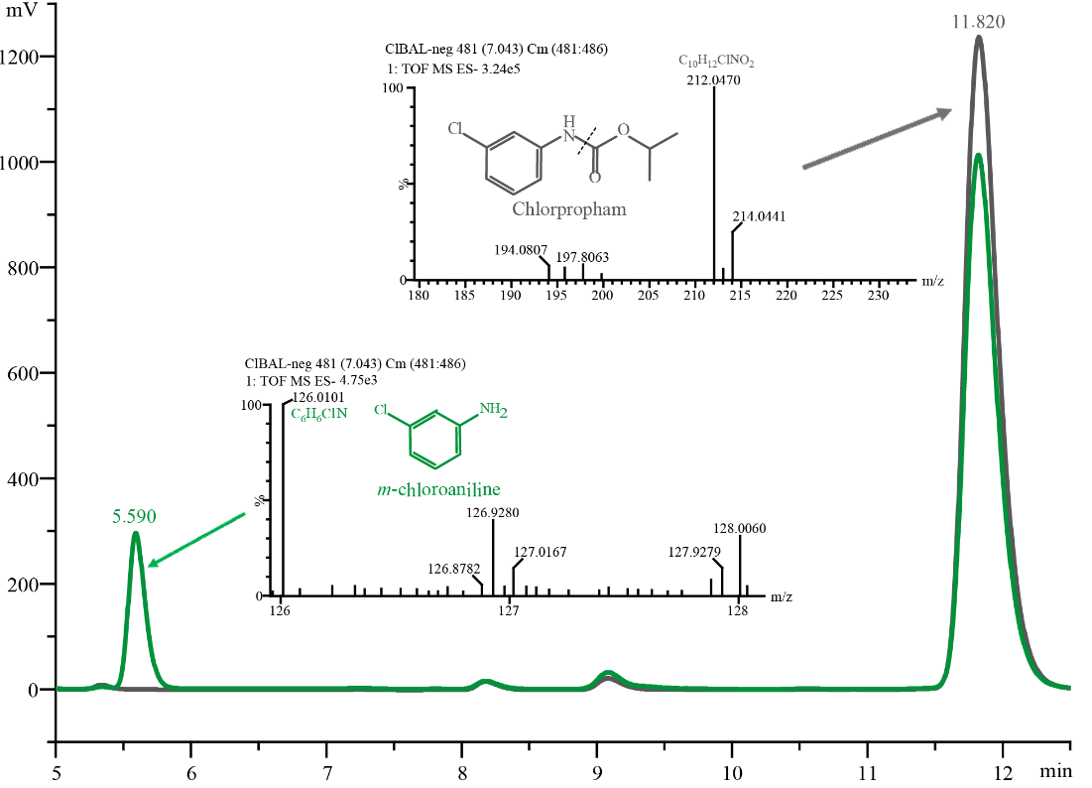
**

**Figure S8.** LC-MS analyses of the metabolite produced during propham degradation by the purified recombinant Ppa. The HPLC profiles of propham [Rt (min)=6.637] and the metabolite aniline [Rt (min)= 5.627] are indicated. The mass spectra of propham {m/z=180.1010 [M+H]^+^} (right) and aniline {m/z=94.0653 [M+H]^+^} (left) are also shown.


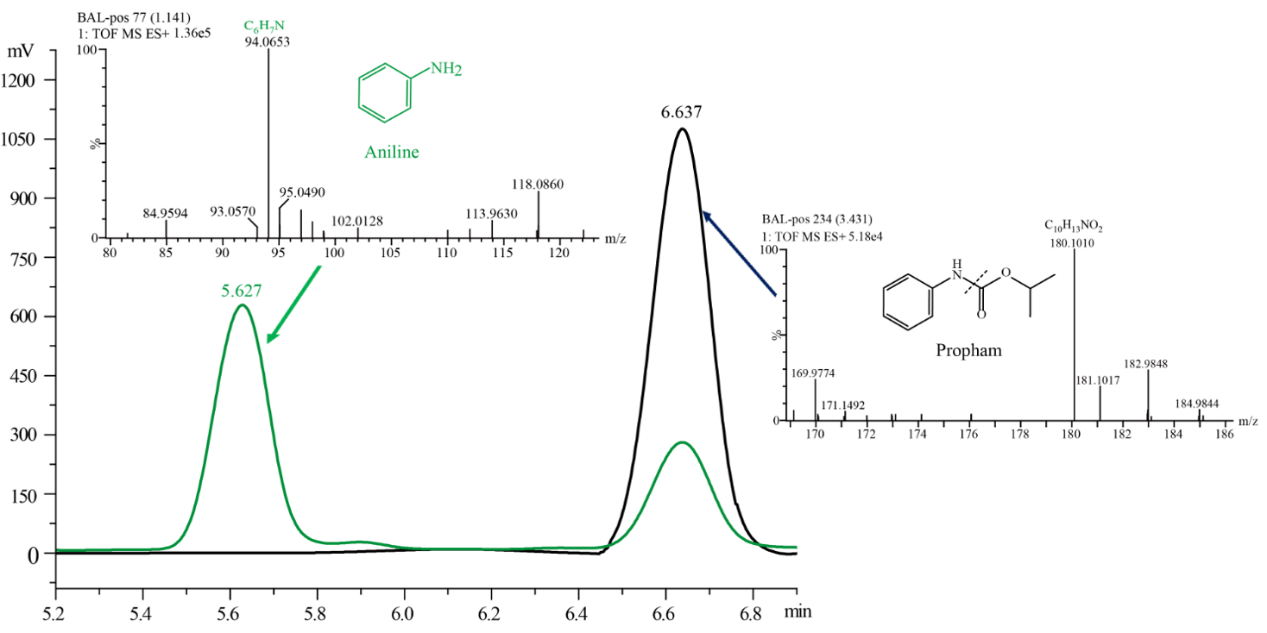


**Figure S9.** Effects of temperature (A), pH value (B) and metal ions (C) on the activities of the purified recombinant Ppa.


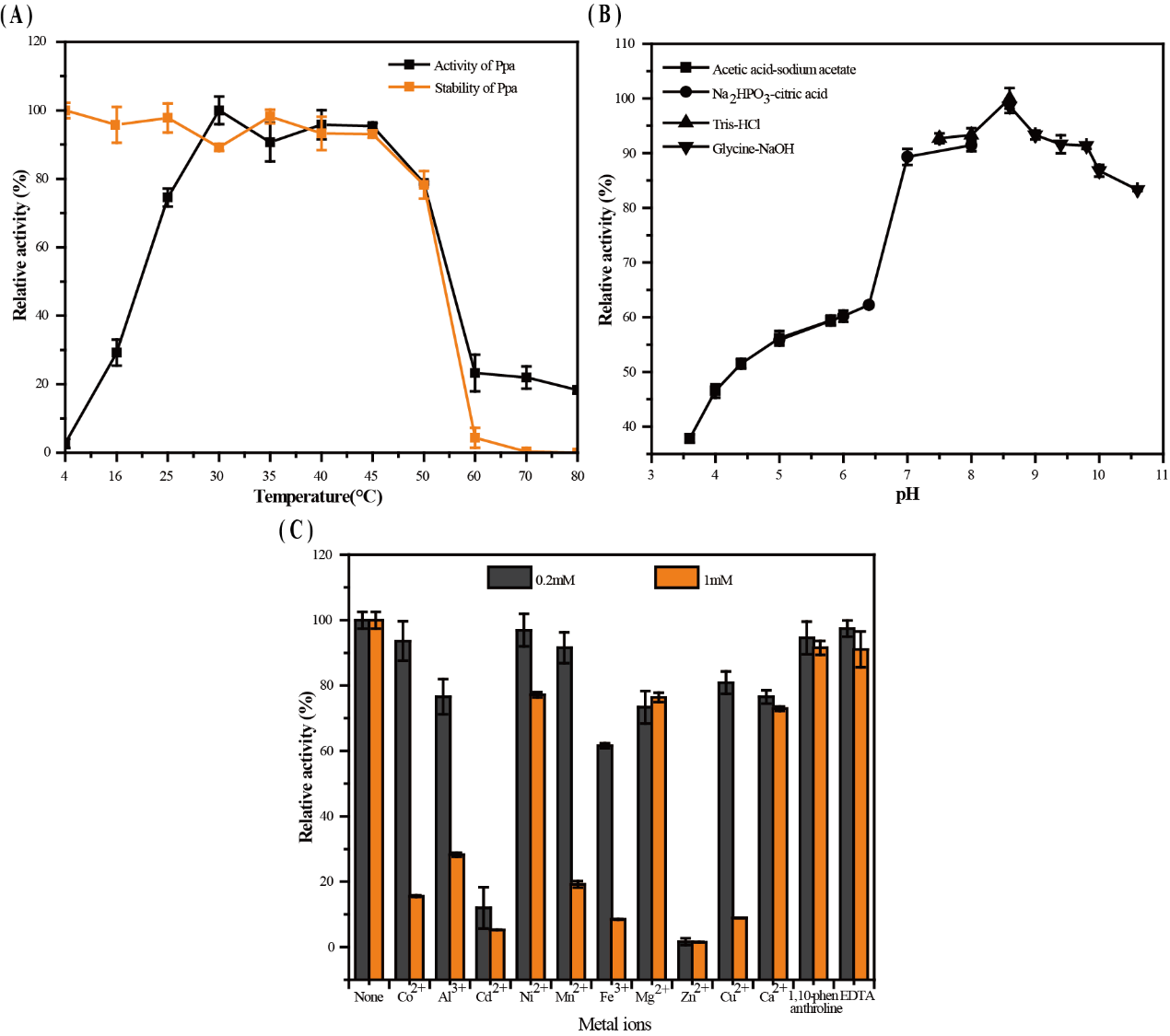


**Table S1 Primers that were used in this study**

| **Function and oligonucleotide** | **Sequence (5’-3’) ^a^** |
| --- | --- |
| **Genetic disruption** |  |
| PP1-F | TAAGGATCCCCAACCCTGCATCGTGAACCTC(*Bam*HI) |
| PP1-R | ATGAACGCACCGGCGCCGTTTTCTGTCTCCCGGCACTGAT |
| PP2-F | ATCAGTGCCGGGAGACAGAAAACGGCGCCGGTGCGTTCAT |
| PP2-R | AAATCTAGAAATTCACATCCCCATCGCTACCAT(*Xba*I) |
| PPA1-F | AGTCTCGAGAGGGGACATTCTAAAGTGG(*Xho*I) |
| PPA1-R | AAGACTAGTGTGAATGGATATGAACGCACC(*Spe*I) |
| **RT-QPCR** |  |
| SWP-3-16S-F | TGATCCAGCAATGCCGCGTG |
| SWP-3-16S-R | ATTTCACCTCCGTCTTACAA |
| SWP-3-PPA-F | GGTCCGACGCGCAATCCATT |
| SWP-3-PPA-R | CCCGGATGGAAAGCATTGG |
| **Gene expression** |  |
| QPP-3-F | AGTAAGCTTCATCAGTGCCGGGAGACAGAA(*Hind*III) |
| QPP-3-R | GTTCTCGAGGTTCGGGCGAACAGGGGCCGTC(*Xho*I) |
| **Random Mutagenesis** |  |
| PP-F | AAAGGGGACATTCTAAAGTGGGCA |
| PP-R | AAATCTAGAGTGAATGGATATGAACGCACC (*Xba*I) |

*^a^* Restriction sites are underlined.

**Purification of Ppa**

The positive recombinants were cultivated in LB broth containing 50 mg/L Km at 37 °C with shaking at 180 rpm. When the OD_600_ reached 0.3-0.4 (approximately 5-6 h), 0.4 mM of isopropyl-*β*-D-thiogalactopyranoside (IPTG) was added, and the cells were cultured at 16 °C with shaking at 160 rpm for 20 h. Cells were harvested (12,000 rpm, 7 min), washed and re-suspended in Tris-HCl buffer (20 mM, pH 8.0) and then lysed by sonication on ice. All purification procedures were performed at 4 °C. Cell debris and insoluble proteins were removed by centrifugation (12,000 rpm, 20 min, 4 °C). The expressed recombinant protein was purified using Ni^2+^-NTA resin [1]. Fractions containing recombinant protein were dialyzed against 20 mM Tris-HCl buffer overnight at 4 °C to remove imidazole. The purity of Ppa was monitored by sodium dodecyl sulfate polyacrylamide gel electrophoresis (SDS-PAGE), and the protein concentration was quantified by the Bradford method using bovine serum albumin as the standard [2].

**Enzymatic characterization of Ppa**

The disappearance of the swep substrate was monitored by HPLC. One unit of enzymatic activity was defined as the amount of enzyme required to catalyze the formation (or hydrolysis) of 1 µmol of product (or substrate) per min. The thermal stability of Ppa was tested by determining the residual activity of the enzyme toward swep after Ppa was preincubated at different temperatures (4-70 °C) for 30 min. The optimal reaction temperature for Ppa was determined by incubating the enzyme at different temperatures (4-70 °C) without preincubation. The optimum pH for enzymatic activity was evaluated at pH values ranging from 3.6 to 10.6 using four different buffering systems: acetic acid-sodium acetate (pH 3.6, 4.0, 4.4, 5.0, and 5.8), Na_2_HPO_4_-citric acid (pH 5.0, 6.0, 6.4, 7.0, and 8.0), Tris-HCl (pH 7.5, 8.0, and 8.6) and glycine-NaOH (pH 8.6, 9.0, 9.4, 10.0, and 10.6). The effects of different metal ions on enzymatic activities were determined by adding different metal ions to the reaction systems at final concentrations of 0.2 and 1 mM for 30 min.

3 mL of Tris-HCl (100 mM, pH 8.0) containing 0.16 µg purified Ppa was incubated with 30 mg/L of different substrates (such as propanil, chlorpropham and propham) at 30 °C for 6 h. The reactions mixtures were subsequently freeze-dried and re-dissolved in 500 µL methanol, with the residues of different substrates evaluated by HPLC. The metabolites produced during the transformation were identified by an ultrahigh pressure LC system (Dionex, Thermo Fisher Scientific) connected to a LTQ Orbitrap XL hybrid mass spectrometer (Thermo Fisher Scientific). For kinetic studies, substrates were appropriately diluted to at least six different concentrations close to the dissociation constant (*K*_m_) values. Kinetic values were obtained using the Hanes-Woolf equation for various substrate concentrations. In all assays, reactions were performed in triplicate [3].

**RT-qPCR**

The contaminating genomic DNA (gDNA) was digested using gDNA Eraser (TaKaRa, China) at 42 °C for 2 min, after which cDNA was generated using 1 g of gDNA-removed RNA, random primers and PrimeScript reverse transcriptase (RTase; TaKaRa). Each sample was diluted to 300 µg/µL and used as template for quantitative PCR (qPCR), which was performed using an Applied Biosystems 7300 real-time PCR system (Applied Biosystems, USA) and a SYBR Premix Ex Taq RT-PCR kit (Tli RNaseH Plus; TaKaRa, China) following the manufacturer’s instructions. The 16S rRNA gene was used as an internal standard, and the relative expression was quantified according to the 2^-∆∆CT^ threshold cycle (CT) method [4].

**References**

1. Janknecht R, de Martynoff G, Lou J, Hipskind RA, Nordheim A, Stunnenberg HG. Rapid and efficient purification of native histidine-tagged protein expressed by recombinant vaccinia virus. Proc Natl Acad Sci. USA. 1991;88:8972-6.
2. Bradford MM. A rapid and sensitive method for the quantitation of microgram quantities of protein utilizing the principle of protein-dye binding. Anal Biochem. 1976;72:248-54.
3. Zhang L, Hu Q, Hang P, Zhou XY, Jiang JD. Characterization of an arylamidase from a newly isolated propanil-transforming strain of *Ochrobactrum* sp. PP-2. Ecotoxicol Environ Saf. 2019;167:122-9.
4. Livak KJ, Schmittgen TD. Analysis of relative gene expression data using real-time quantitative PCR and the 2(-Delta Delta C(T)) Method. Methods. 2001;25:402-8.
